# Constructing validity evidence from a pilot key-features assessment of clinical decision-making in cerebral palsy diagnosis: application of Kane’s validity framework to implementation evaluations

**DOI:** 10.1186/s12909-023-04631-4

**Published:** 2023-09-14

**Authors:** LM McNamara, KM Scott, RN Boyd, EA Farmer, AE Webb, IE Novak

**Affiliations:** 1 Specialty of Child and Adolescent Health, Faculty of Medicine, The University of Sydney, Sydney, Australia; 2 The Faculty of Medicine, Queensland Cerebral Palsy and Rehabilitation Research Centre, The University of Queensland, Brisbane, Australia; 3 Graduate School of Medicine, University of Wollongong, Wollongong, Australia; 4 Faculty of Medicine and Health, Cerebral Palsy Alliance Research Institute, The University of Sydney, Sydney, Australia; 5 Faculty of Medicine and Health, The University of Sydney, Sydney, Australia

**Keywords:** Key-features assessment, Early diagnosis, Cerebral palsy, Clinical decision-making, Validity argument, Implementation

## Abstract

**Background:**

Physician decision-making skills training is a priority to improve adoption of the cerebral palsy (CP) clinical guideline and, through this, lower the age of CP diagnosis. Clinical guideline implementation aims to improve physician practice, but evaluating meaningful change is complex. Limitations in the validity evidence of evaluation instruments impact the evidence base. Validity frameworks, such as Kane’s, enable a targeted process to gather evidence for instrument scores, congruent to context and purpose. Yet, application of argument-based methodology to implementation validation is rare. Key-features examination methodology has established validity evidence supporting its use to measure decision-making skills, with potential to predict performance. We aimed to apply Kane’s framework to evaluate a pilot key-features examination on physician decision-making in early CP diagnosis.

**Methods:**

Following Kane’s framework, we evaluated evidence across inferences of scoring, generalisation, extrapolation and implications in a study design describing the development and pilot of a CP diagnosis key-features examination for practising physicians. If found to be valid, we proposed to use the key-feature scores as an outcome measure of decision-making post education intervention to expedite CP diagnosis and to correlate with real-world performance data to predict physician practice.

**Results:**

Supporting evidence for acceptance of scoring inferences was achieved through examination development with an expert group (*n* = 10) and pilot results (*n* = 10): (1) high internal consistency (0.82); (2) acceptable mean item-discrimination (0.34); and (3) acceptable reliability of examination scorers (95.2% congruence). Decreased physician acceptance of examination time (70%) was identified as a threat and prioritised in case reduction processes. Partial acceptance of generalisation, extrapolation and implications inferences were defensible with: (1) accumulated development evidence following established key-features methodology; (2) high pilot acceptance for authenticity (90%); and (3) plausibility of assumptions of score correlation with population register data.

**Conclusions:**

Kane’s approach is beneficial for prioritising sources of validity evidence alongside the iterative development of a key-features examination in the CP field. The validity argument supports scoring assumptions and use of scores as an outcome measure of physician decision-making for CP guideline education implementation interventions. Scoring evidence provides the foundation to direct future studies exploring association of key-feature scores with real-world performance.

**Supplementary Information:**

The online version contains supplementary material available at 10.1186/s12909-023-04631-4.

## Introduction

Expediting evidence to practice continues to be a complex challenge for health professions education and health care systems. An average clinical practice time lag of 17 years has been widely reported [1, 2]with approximations of 9.3 years from publication to practice implementation [1]. The impetus for evidence-informed practice is reflected in the growth of new research fields targeting the evidence to practice gap in the past two decades, such as implementation research [3–5]. Implementation science seeks to modify clinical practice, behaviour or policy and increase the use of evidence-based practice [6]. This emerging field can include educational interventions and may provide opportunities for convergence research with health professions education [7] to accelerate translation of evidence into routine clinical practice.

Limitations in the validity evidence of implementation evaluation instruments have been identified, warranting further investigation [8]. Testing and evaluating theories that underpin intervention development and evaluation is fundamental to implementation science [9–14], however there is little application of validity theory [15–17] to evaluation instruments [17, 18]. Contemporary validity frameworks such as Kane’s can guide use of validity testing theory through the collection of priority evidence according to assumptions of how scores will be used and in what context [19–23]. Kane’s framework involves two interconnected arguments: (1) an interpretative and use argument for test scores; and (2) a validity argument evaluating the plausibility of interpretations and use [19]. The interpretative argument includes specified inferences and assumptions that lead from test performances to real-world score-based implications. The chain of inferences from scoring (measurement of performance as a score), generalisation (scores reflecting test setting performance), extrapolation (scores reflecting real-world performance) and implications (score application to individual outcomes) creates a framework for validation of a presumptive argument [20, 21]. The validity argument evaluates the inferences, seeking to establish if assumptions are demonstrably plausible through supporting evidence [20, 21]. Use of contemporary validity approaches are rare in health professions education evaluation [24–28] despite recommendations from field leaders [25, 29]. The paucity of application of validity frameworks in implementation research warrants further attention.

To address this gap, we applied Kane’s framework to the development of an outcome measure for a tailored implementation intervention targeting an identified research-practice gap in the field of cerebral palsy (CP), the most prevalent motor disability in childhood [30]. Early, accurate CP diagnosis before six-months of age is possible using predictive clinical assessments and clinical decision-making skills [30]. Yet population registers indicate a CP diagnosis typically occurs between 12–24 months of age in high-income countries, with a median age of three years in low-income countries, suggestive of a ‘wait and see’ approach to CP diagnosis in clinical practice [30–32]. Implementation interventions to expedite a clinical diagnosis under six-months of age are an identified priority for health professions education [33]. A tailored online implementation intervention has been developed targeting physician diagnostic behaviours and clinical decision-making skills in the early diagnosis of CP [34]. This study explored validity evidence of scores from a CP key-features examination for use as a post-intervention outcome measure of physician clinical decision making.

The key-features approach to assessment measures a clinician’s essential clinical decision-making skills [35]. Key-features are case specific and determined by a consensus process with clinical experts [35, 36]. Testing only the critical elements of a problem, labelled as key-features, contributes to reduced testing time on unnecessary areas of a problem and a larger number and range of clinical problems in an examination [35]. In non-CP populations, key-feature cases have established validity evidence to measure the construct of clinical decision-making if robustly designed [37]. Moreover, summative examination scores have been demonstrated to predict future practice in physicians [38–40].

**Fig. 1:**
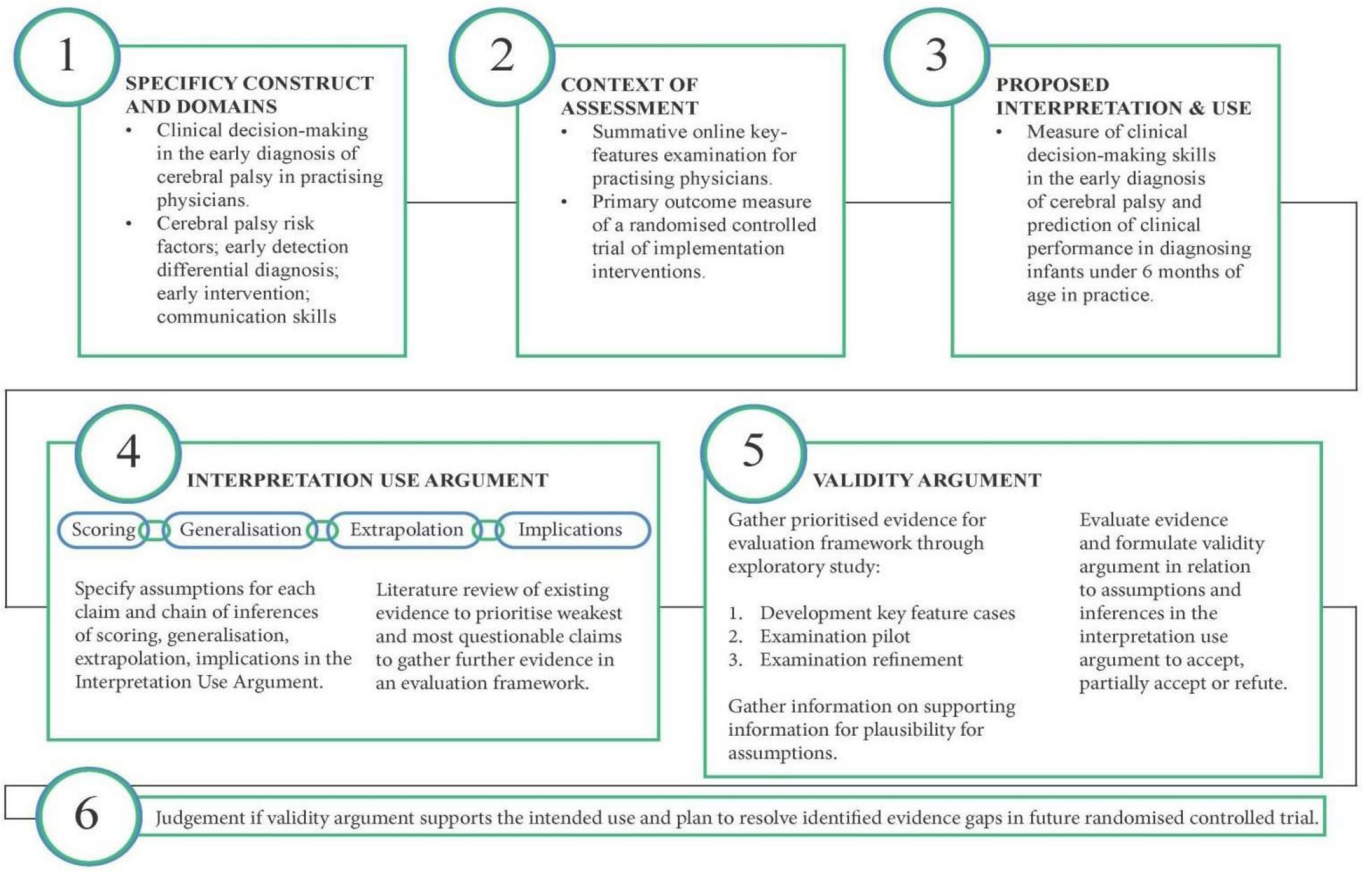
Overview of study using Kane’s framework of validity

This paper describes: (1) the development and pilot of a web-based key-features examination for practising physicians; and (2) Kane’s validation approach of an interpretation use argument for examination scores and evaluating evidence of inferences in a validity argument. An overview of Kane’s validity framework is displayed in Fig. 1.

## Method

### Construct and context

We developed a web-based key-features examination to measure clinical decision-making skills in the early diagnosis of CP in practising physicians. The examination was developed so that post-intervention online key feature examination scores could be used as an outcome measure of decision-making skills in a randomised controlled trial (RCT) of e-learning for practising paediatricians in the Australian context. The strength of association of physician key-feature case scores with real-world patient outcomes will be explored through correlation with population CP registers to predict physician performance in diagnosing CP under six-months of age in clinical settings.

### Defining the interpretive and use argument

Based on our proposed use of key-feature examination scores, we articulated assumptions in the interpretative use argument according to Kane’s four-stage chain of inferences [20, 21]. Kane describes rules for making warrants that require backing or evidence [21]. We described a warrant for each inference and made assumptions for each warrant. In Kane’s framework, qualifiers are a form of rebuttal which can indicate uncertainty of assumptions [21]. We considered qualifiers that may weaken the plausibility of our assumptions and appraised the established validity evidence of the key-features approach to further guide the collection of evidence required to support our claims. Table 1 summarises the initial inferences, warrants, assumptions, evidence, and qualifiers adapted from Kane’s generic network of inferences [21] for the purposes of this study.

**Table 1:** Summary of inferences, warrants, assumptions, evidence, and qualifiers (Kane 2013) [21] for the use of key-feature cases and scores

Inference	Definition	Warrant	Assumptions	Backing/Evidence	Rebuttal/Qualifier
Scoring	Refers to the steps involved in taking an observation of performance to an observed score; it looks at scoring rules, rubric and scoring procedures (Kane 2013) [21]	Observation of candidate’s performance on test reflects a measure of clinical decision-making skills in early diagnosis of cerebral palsy that is appropriate, accurate, free of bias	1) The construct measured by the key-features examination is clinical decision-making 2) Online testing conditions are standardised 3) The scoring rubric and scoring conditions are free of bias and function as intended 4) The items demonstrate adequate psychometric qualities and test functions as developers intended	Content evidence demonstrates key constructs are identified through task analysis with experts. Appraisal of existing evidence to guide standardised test development procedures, testing conditions and scoring rubrics. Sources of evidence through piloting to support or refute warrants; feasibility of testing conditions, psychometric test item qualities and whole test, reliability of scoring.	Certain contextual factors are not modelled in the test tasks
Generalisation	Refers to the extent to which a single test score represents potential performances in the domain and context (Kane 2013) [21]	The selection of item sampling representative of the relevant test domain	1) Number of key-feature cases provides a reliable estimate of candidate performance 2) Key-features are representative of the examination blueprint 3) Key-feature case scores are influenced by prior clinical decision-making skills training in the early diagnosis of CP	The selection of item sampling is representative of the relevant test domain. Blueprint representation of content mapped to domain. Scoring keys are reliable for masked assessors. Review of literature for key-features reliability. Pilot study of development examination for reliability.	Test score reliability is low. Scorer inter-rater reliability is low. Acceptance of physician users for length of key-features examination for adequate sample of cases. Different group of test takers perform differently on the same test. Item bias of domain found.
Extrapolation	Refers to evidence for expected future performance in different clinical contexts (Kane 2013) [21]. Represents the quality of performance in the real-world.	Test scores reflect actual performance in the early diagnosis of cerebral palsy	1) Key-feature cases test the skills essential to physician clinical decision-making in the early diagnosis of CP in a clinical setting 2) Key-feature cases are authentic representations of real-world cases 3) Key-feature case scores differentiate levels of expertise in the early diagnosis of CP 4) Key-feature case scores are associated with clinical performance in diagnosing infants with CP under 6 months of age	Key-features represent skills related to construct. Key-features and scoring rubrics accurately demonstrate difference in performance levels on the construct in response to training and identify appropriate external criterion to compare with performance on test.	Performance on test is not related to clinical decision-making skills. External criterion is not reliable or valid for score interpretation or uses. Consideration of contextual differences between states and territories. Requires extensive barrier analysis. Ceiling effect observed in scores of candidates who participate in the randomised controlled trial due to prior implementation interventions.
Implications	Refers to the use of scores for decisions about individuals’ outcomes and implications of those decisions (Kane 2013) [21]	The property of scores supports the implications and real-world consequences	1) Key-feature case scores will be associated with adherence to a clinical guideline in the early diagnosis of CP 2) Key-feature case scores will be associated with patient outcomes of early intervention and parent supports 3) Completion of key-features examination will create challenges that drive learning	Consequences evidence on patient outcomes-funding packages received under 6 months of age. Correlation analysis. Measures of behaviours and readiness using theoretical framework. Consequences evidence on candidates with study burden and incentives. Consequences evidence on impact on candidate of completing the examination.	Consideration to be given to other moderators and facilitators of behaviour change in clinical practice. Individual constructs such as motivational factors, self-efficacy, theory of planned behaviour. Ceiling effect is observed in scores of candidates who participate in the randomised controlled trial due to prior implementation interventions.

## Appraisal of existing evidence according to Kane’s chain of inferences

### Scoring

Two previous reviews support the key-features assessment measuring the construct of clinical decision-making [37, 41]. Bordage outlines the body of convergent [42–44] and divergent evidence [42, 45–47] indicative of key-feature cases measuring complex and elaborative cognitive processes as opposed to simple knowledge constructs.

Evidence supporting scoring assumptions of internal consistency reliability and test item discrimination can be collected through robust development and piloting phases [42, 48]. An internal consistency level of Cronbach’s alpha of 0.8 or higher is preferable as evidence of reliability [41]. Acceptable reliability Cronbach’s alpha levels between 0.7 and 0.9 have been reported with longer examinations of between 25–40 cases [41]. Bordage and Page [37] emphasise the homogeneity of the group of candidates and discriminating ability of key-features may also impact score reliability and the amount of cases required to differentiate. Of note, Trudel et al [42] reported high levels of reliability and demonstrated differentiation with 9 key-feature cases between general and subspecialty physicians. Item discrimination levels above 0.30 are recommended with regards to scoring evidence assumptions [49, 50].

Existing recommendations to optimise reliability and internal consistency support: the use of short menu and write-in responses [51]; lay language in scenarios [52]; cases with 2–3 key features rather than single questions [37, 53]; using the case not the key-feature question as the unit of examination measurement [35]; equal weighting for key-features within each case [35]; and selection of cases based on information about their discrimination level [41]. The use of ‘write-in’ responses for diagnosis can assist in discrimination with weaker candidates [37].

Although the key-features approach is more frequently applied to high-stakes examinations [37], previous studies have applied summative examination scores in the context of continuing medical education [43]. Doucet compared two continuing medical education formats with practising physicians for headache diagnosis and management [43]. A 60-min examination 3-months post-intervention was able to demonstrate a 25% difference in mean key-feature examination scores favouring the intervention group [43]. To date, the key-features approach has not been applied in the field of CP.

Acceptance of web-based testing conditions for an implementation intervention is also required to support scoring assumptions in our argument. Web-based testing conditions have been explored in practising physicians and need to be considered for construct-irrelevant variance [42]. Scoring rubric construct irrelevant variance may be reduced with an expert panel consensus process as described in previous examples of pilot testing [42, 48, 54]. Reliability of scoring rubrics and examination scorers needs to be demonstrated in a strong argument to be free of bias and function as intended, in particular for the testing of communication skills when delivering a diagnosis with no comparative examples in the literature.

### Generalisation

Generalisation inferences refer to the degree to which a single examination score represents all possible performances in the test domain and context [21]. Generalisation assumptions necessitate item sampling representative of the test domain and require thorough domain-test blueprinting as demonstrated by numerous authors [42, 48, 55, 56]. Demonstration of reliability through Cronbach’s alpha can also strengthen arguments for generalisation assumptions. Evidence for relationship to other variables can be explored through piloting phases, in particular the influence of clinical experience, levels of expertise and prior training on examination, which may also impact the strength of extrapolation inferences [42, 57].

### Extrapolation

Strong evidence is required in our validity argument to support extrapolation claims of associations of key-feature examination scores with clinical performance and patient outcomes. No studies exploring the correlation of key-feature examination scores post-educational intervention with impact on future practice or patient outcomes were identified in previous reviews. Tamblyn et al., [38, 40, 58] however, provides evidence to support the predictive validity of high-stakes key-feature case examination scores through correlations with rates of complaints to medical regulatory bodies [38, 40] and patient adherence to anti-hypertensive treatment [40]. Examples of evidence supporting authenticity of key-feature cases as representations of real-world cases is established in the literature but necessitates supportive evidence through consensus development phases and piloting for user acceptance [42, 48, 59].

### Implications

Implications evidence is less frequently reported in key-features literature, aligning with validity evidence gaps previously identified in health professions education [60, 61]. The burden on test developers has been identified [42] but warrants further investigations to assess validity and feasibility when considering developers’ time, costs, and consequences. Schuwirth [62] estimated development of an individual key-feature case takes up to 3 h for experienced teams. Evidence supporting the impact on learners to support extrapolation and implications inferences should be considered in a validity argument, including the consideration of formative assessment for learning.

## Interpretation use argument

In developing an interpretation use argument we considered assumptions of inferences that could be identified a priori on the basis of existing evidence or following established guidelines and those that could be achieved through examination development and piloting phases. For stronger assumptions, such as correlation with external criteria, we have specified the research methodology and other sources of evidence required in future validation studies to support these claims.

Determining the strength in the association of examination case scores with other related measures of clinical performance requires plausibility in assumptions and validity evidence of the associated measure. We have identified two population-based CP registries for the purpose of correlation with physician key-feature case scores in future validation studies. Each Australian state and territory has a CP register, with data aggregated into one single population register. Registration is offered to parents after a clinical diagnosis of CP is confirmed or taken as a mandatory report under the public health act. Australia’s National Disability Insurance Agency (NDIA) provides funding for parents to buy early intervention for eligible children with a clinical CP diagnosis. Physician referrals to these national CP datasets have been included in the evaluation framework of the RCT to enable correlation of association with key-feature case scores [34].

The interpretation use argument and evaluation framework outlining the underpinning assumptions, research questions and type of validity evidence required to support the validity argument is outlined in Table 2.

**Table 2:** Interpretation use argument assumptions [21] organised by each level of inference, research questions to address assumptions, sources of evidence required for validity argument and pilot study hypotheses

Interpretation/Use Argument Assumptions	Research questions to answer assumptions	Sources of evidence required for validity argument	Hypotheses for pilot study
The items and test exhibit good psychometric qualities and test functions as developers intended	*Prioritised Research Question* (1) What is the internal-consistency reliability of the scores from the key-features examination? *Prioritised Research Question* (2) How discriminating are the key-feature cases?	Pilot study reliability analysis	*Hypothesis* (1): Final examination case scores will demonstrate high internal consistency reliability Cronbach’s alpha > 0.65 *Hypothesis* (2): Final examination case scores will differentiate between high- and low-scoring examinees (2.1) Case item- difficulty between 0.2 and 0.8 (2.2) A positive score in item-total correlation (2.3) Mean item- discrimination indices of at least + .3.0 or higher
Online testing conditions are standardised	*Prioritised Research Question* (3) What is the acceptability of an online examination using the key-features approach for paediatric physicians?	Pilot study user acceptance	*Hypothesis* (3): (3.1) Online examination using the key-features approach will be acceptable for paediatric physicians (3.2) Time taken is acceptable for practising physicians
Scoring rubric and scoring conditions are free from bias and function as intended	*Prioritised Research Question* (4) What is the reliability of examination assessors?	Pilot study expert consensus on scoring rubric and high scorer reliability between masked assessors and research group	*Hypothesis* (4): Total congruence between masked assessors and research group will be > 95%
Number of key-feature cases provide a reliable estimate of candidate performance	What is the internal-consistency reliability of the scores from the key-feature examination?	Pilot study reliability and correlation analysis	
Key-features are representative of the examination blueprint	What is the relative distribution of cases and key- features according to the blueprint and priority behaviours?	Mapping and % distribution of cases according to blueprint and top three priority behaviours in development group process, pilot study and refinement of final examination	
Key-feature case scores are influenced by prior clinical decision-making skills training in the early diagnosis of CP	To what extent are the case scores affected by examinee practice type, prior training in the early diagnosis of CP and years of experience in the early diagnosis of CP?	Pilot study baseline demographics will be collected for all study participants including workplace, profession, proportion of CP caseload, number of years of clinical experience diagnosing CP, awareness of early diagnosis clinical practice guideline, prior training in the early diagnosis of CP, GMA, and HINE	
Key-feature cases assess the skills essential to physician clinical decision-making in the early diagnosis of CP in a clinical setting	To what extent do experts in the development group agree on key-features?	Expert advisory group consensus on key-features in development phase	
Key-feature cases are authentic representations of real-world cases	How authentic are the cases in the examination?	Expert advisory group consensus in development phase. Pilot study user acceptance data	
Key-feature case scores differentiate levels of expertise in the early diagnosis of CP	How discriminating are the key-feature test items?	Pilot study mean case scores according to awareness of clinical guideline, clinical caseload, and prior training	
Key-feature case scores will be associated with clinical performance in diagnosing infants with CP under 6 months of age	To what extent are candidate case scores related to external criteria of candidate clinical performance measures of diagnosis of CP?	Future randomised controlled trial analysis of correlation between case scores and external criterion over six-month study duration. Number of physician referrals under six-months of age to the CP Register. Number of physician access requests under six-months of age to the National Disability Insurance Agency.	
Key-feature case scores will be associated with patient outcomes of early intervention and parent supports	To what extent are candidate case scores related to external criteria of patient outcomes of early intervention and parent supports?	Future randomised controlled trial analysis of correlation between key-feature examination case scores and external criterion over six-month study duration. Number of physician access requests under six-months of age to the National Disability Insurance Agency. Number of eligible participants entered into the National Disability Insurance Scheme under six-months of age.	
Completion of the examination will have consequences for examinee candidate in creating desirable difficulties and driving learning	What is the impact on examinee on completing the test activity itself on driving learning?	Pilot study user acceptance feedback	

## Exploratory study of key-features examination development and piloting

**Fig. 2:**
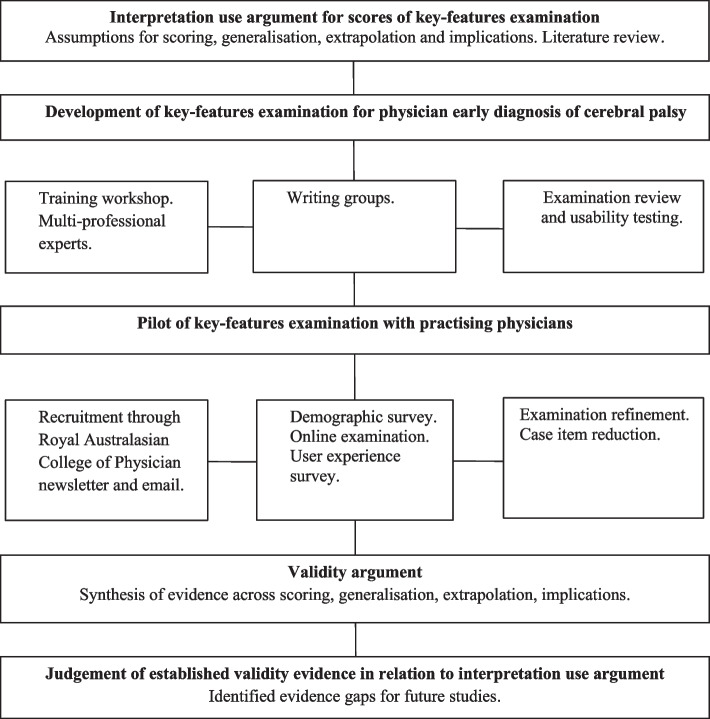
Flow diagram of exploratory study

The purpose of the exploratory study was to: (1) repurpose the key-features approach to assessment for practising physicians in the field of CP; and (2) evaluate the validity evidence of key-features examination scores. The study was comprised of three phases: (1) *Development* of a web-based key-features examination with an expert advisory group supervised by a key-features field leader (EF); (2) *Pilot* of the examination to determine internal-consistency, item discrimination, acceptance with practising physicians, and reliability of examination scorers; and (3) *Refinement* of the final examination. Our hypotheses for the pilot study given our intended interpretation and use of examination scores are provided in Table 2 and were prioritised by our Interpretation Use Argument assumptions. A study flow diagram is provided in Fig. 2.

### Ethics

The study received ethical approval from The University of Sydney Human Research Ethics Committee (Project number 2020/008).

### Development group participants and recruitment

Examination development was conducted between June 2020 and March 2021. The key-features development group comprised a 10-member expert panel involving experts in the early diagnosis of CP (*n* = 8), medical education (*n* = 1) and the key-features approach (*n* = 1). The early diagnosis of CP experts were purposely selected from the authors of the CP guideline [30] and clinical leaders from across Australia and Italy. Potential participants were invited to participate via email.

### Development procedure

The methodology for examination development followed published key-feature item writing guidelines [35, 54, 63]. Key-feature writing training was facilitated by EF.

The examination blueprint and weighting of domains were created by the research and advisory group via: (1) review of published practice guidelines for the early diagnosis of CP [30, 64, 65]; (2) a research prioritisation process of key target behaviours for paediatric physicians requiring practice change [33]; and (3) cognitive task analysis [66] of constructs in the tailored implementation intervention.

Expert advisory group participants were invited to complete an 8-question demographic survey and attend an online training workshop on the key-features approach. Participants were provided with online resources prior to the training workshop including: video resource lectures and PowerPoint presentations on the conceptual background to the key-features approach and key-feature case writing (available by contacting corresponding author), key-feature writing fact sheets (Supplementary Material File 1), writing and scoring templates (Supplementary Material File 2), examination blueprint and implementation intervention content.

Participants were provided with additional information regarding question formats and scoring keys during the training workshop. Two question formats of ‘short-menu’ (select from a prepared list) or ‘write-in’ responses (answer given in short note format) were used. Scoring involved a case score, ranging between 0 and 1, which was comprised of the average of the key-feature questions within each case. Each key-feature was weighted evenly. A total examination score was achieved by the sum of the case scores.

During the training session participants were assigned writing partners and broken into writing groups. Each writing group was asked to define key-features for a problem from an examination blueprint domain, source relevant references for key-features and write key-feature cases. Small group writing sessions were followed by whole group discussions for panel agreement on key-features and critical actions of each case. Following the training workshop, writing partners were asked to write a minimum of two further key-feature cases within an 8-week time frame. Authors (LM, EF) facilitated an iterative process via email with panel members to review cases and scoring keys and obtain consensus on key-features. The development group wrote a total of 21 cases with 2–5 key-features per case (sample case in Supplementary File 3). There were 51% of ‘write-in format’ key-feature questions and 49% of ‘select from list’ format.

The final 63 key-feature questions and scoring keys were reviewed by the research group and two expert group members (paediatrician, paediatric neurologist) to achieve final agreement that key-features assessed critical areas of the case and about wording of questions, scoring keys and authenticity of scenarios. Disagreements were resolved via email. ‘Write-in’ responses for questions assessing communication skills required the greatest number of revisions of scoring keys.

Examination instructions were developed, including downloadable fact sheets and video instructions. Instructions included key-features assessment tips, scoring information, and examples of ‘select from list’ and ‘write in’ question formats. Instructions were piloted (*n* = 3) to affirm comprehensibility. The examination was accessible via a REDCap [67] survey link. Each question could only be answered once, and no backward navigation was possible to view or change previous answers.

Usability of the 21-case examination was tested using a think-aloud process [68] with an expert in medical education and paediatrician. Minor revision of case wording, examination instructions and REDCap formatting was completed and the examination case sequence was re-ordered. An estimated average total test time of 90-min was taken from field testing with health professionals in clinical practice (*n* = 3).

### Pilot group participants and recruitment

Pilot group participants were practising paediatric physicians recruited from across Australia. Participants were invited to participate via an advertisement in the Royal Australasian College of Physicians newsletter and email distribution of opinion leaders in the early diagnosis of CP.

### Pilot procedure

Examination piloting was conducted between April and June 2021. Participants were invited to complete a 10-question demographic survey prior to commencing the online examination. They were provided with information about the examination procedure, scoring and testing conditions. Specifically, participants were asked to complete the examination under closed-book testing conditions and told the anticipated time to completion was 90-min. Participants were asked to confirm that they had read the examination instructions and agreed to closed-book testing conditions. Key-feature cases were named, and a progress bar was provided throughout the examination. An email reminder was sent up to three times for participants who partially completed the examination. Upon completion of the examination, participants were invited to complete a 9-question user experience survey. User acceptance questions developed by Bronander et al [59] were adapted for this study.

### Analysis and scoring

Examinee responses were de-identified. An initial scoring meeting was conducted with the research group to review score reports, scoring keys and write-in responses. Each case was reviewed for clarity to gauge if the question was behaving as intended from examinee comments. Refinements to scoring keys and instructions for scorers were actioned.

A masked assessor, independent of the research group, conducted scoring on all cases. The masked assessor was an experienced clinician in early CP diagnosis and was provided with a training session on the scoring keys and key-feature fact sheet training resources. A second scoring meeting was conducted with the masked assessor and research team, further refinements were made to scoring keys, and a final round of scoring was conducted by the research group and masked assessor.

Descriptive statistics were used to analyse the score distribution on demographic and user acceptance surveys. User acceptance questions comprised five-point Likert scale answer options. The analysis combined the responses ‘strongly agree’ with ‘agree’ and ‘strongly disagree’ with ‘disagree.’ For the question comparing the difficulty of the key-feature examination to a standard multiple-choice examination, the analysis combined ‘much more difficult’ with ‘more difficult’ and ‘less difficult’ with ‘much less difficult.’ Internal consistency was calculated from case scores using Cronbach’s alpha. Case difficulty was estimated from mean averaged case scores. Case score item-discrimination and inter-item total correlation were calculated. A factor analysis was not anticipated due to the small sample size of the pilot but would be considered after inspecting the correlation matrix for a correlation coefficient over 0.30.

## Results

### Participant characteristics

Twenty-eight participants completed the demographic survey. Nineteen participants commenced the key-features examination; however, data from 9 participants are not included in the main analysis as they did not complete the examination, resulting in 10 records for descriptive and correlation analysis. All these 10 participants completed the user experience survey. Overall, the majority of participants identified as paediatricians (80%), had ten or more years’ experience in CP diagnosis (60%), worked clinically in a hospital setting (90%), and less than 10% of their caseload comprised CP patients (70%). The majority of participants reported awareness of the Novak et al [30] guideline (60%); 30% of participants had completed accredited courses in recommended CP diagnostic tests (Prechtl’s General Movements Assessment [69] and the Hammersmith Infant Neurological Examination [70]) [30]. The demographic details of participants are presented in Supplementary Material File 3, Table 1.

### Content related to blueprint

The cases and key-features of the pilot examination of 21 cases were mapped to the examination blueprint (Supplementary Material File 3, Table 2). Cases and key-features tested from more than one domain of the examination blueprint, which is representative of the pooled diagnostic accuracy of two or more tests in clinical practice [71].

### Internal structure and reliability

The reliability coefficient (Cronbach alpha) was 0.83 on the 21-case examination. Mean inter-item correlation was 0.21 and mean item-discrimination was 0.24 (SD = 0.15). The average mean score was 0.56 (SD = 0.24). A factor analysis was not possible with a small sample size and mean inter-item correlation was less than 0.30. Table 3 presents the descriptive statistics for the 21-case examination.

**Table 3:** Descriptive statistics for pilot study (*n* = 21 key-feature cases)

Case Number	1	2	3	4	5	6	7	8	9	10	11	12	13	14	15	16	17	18	19	20	21
Item-Discrimination	0.48	0.18	0.41	0.22	0.44	0.44	0.15	0.28	0.06	0.11	0.00	0.21	0.32	0.09	0.52	0.06	0.15	0.25	0.19	0.31	0.08
Mean	0.75	0.43	0.46	0.39	0.79	0.44	0.82	0.71	0.63	0.63	0.52	0.63	0.41	0.57	0.62	0.40	0.38	0.66	0.47	0.63	0.42
Standard Deviation	0.30	0.15	0.26	0.14	0.22	0.26	0.12	0.25	0.11	0.13	0.21	0.15	0.18	0.21	0.27	0.29	0.16	0.13	0.23	0.23	0.24

### Relationship to other variables

No statistical analysis was performed due to the small sample size; however mean scores according to awareness of guidelines, clinical caseload, and prior training in the early diagnosis of CP recommended tests were reviewed (Supplementary Material File 3, Table 3).

### Format

The scaled average score for ‘write-in’ format questions was 0.56 and for ‘select from list’ was 0.55. The mean item discrimination for ‘write-in’ format questions was 0.32 and for ‘select from list’ was 0.18. Most participants chose to answer ‘write-in’ format questions relating to communication skills in longer sentences despite examination instructions specifying a succinct few words or short phrases, warranting further investigation of this domain.

### Scorer reliability

Total congruence with the masked assessor after round one scoring was 88.3%, and after scoring key refinement process, round two scoring was 95.2%.

### Time taken

Only one participant completed the examination in under 90-min (85-min). Average mean case or question time was not able to be calculated for all participants with accuracy as absence from the platform could not be accounted for.

### User acceptance

There was strong agreement (70%) that the examination and scoring instructions were clear. Authenticity was well supported, with 90% of participants agreeing that cases resembled problems from clinical practice. The majority of participants (70%) reported that the time taken to complete the examination was not acceptable. In comparing the format to a multiple-choice examination, most participants (60%) reported no difference in difficulty.

### Feedback

In free-text responses in the user experience survey about the online testing format and unsupervised conditions, there were six comments in total, all in favour of the online testing conditions. Participants described the clarity and flow of the examination, the interesting variety of cases that were similar to patients they saw in practice, and the learning value of the examination as aspects they liked best: “*The range of problems highlighted what I need to learn more about*.”

In regard to aspects they would most like to change about the examination, the length of the examination was highlighted by four participants. One participant identified a lack of immediate feedback, and that question complexity was difficult for a general paediatrician. One participant advised that the use of the words ‘investigation’ and ‘assessment’ may be misread in questions. Participant feedback responses and pilot data were used to enhance further development of the examination.

### Refinement of the final examination

Reducing the time burden for physicians in practice was prioritised in the refinement phase to enhance acceptance along with exploration of questions or cases for sources of irrelevant variance. A further review of language was conducted for clarity and appropriateness, with particular attention to questions assessing communication skills.

### Case item reduction

Initial reviews focused on case scores with item-discrimination values under 0.2 [50], key-feature questions with negative item-discrimination scores [37], item total-correlation scores 0.8 or higher, key-features targeting the same domains or repetition in type of question. Ten cases were removed. Of the remaining 11 cases, one case with item-discrimination below 0.2 was retained as there was consensus that the case was a priority as it tested a CP differential diagnosis key-feature question not tested in any other case. All 11 cases underwent a further review of key-feature question item-discrimination and mapping to the blueprint.

Descriptive statistics for a final examination of 11 cases and 27 key feature questions demonstrated reliability with Cronbach’s alpha 0.82, mean inter-item correlation of 0.30, and an average mean score of 0.54 (SD = 0.28). We estimated the length of examination time as under 1 h. This was confirmed with 3 practising clinicians. The distribution of the 11 cases mapped to the blueprint is described in Supplementary Material File 3, Table 4.

**Table 4:** Validity argument supporting evidence for chain of inferences of scoring, generalisation, extrapolation and implications [21]

**Supporting evidence for study research questions to answer interpretation/use assumptions **	**Interpretation/ Use Argument Assumptions (as identified in **Table 2**.)**			
	**SCORING INFERENCES** Accepted (✓) Partially Accepted (?) Not Accepted (x)			
	**1**	**2**	**3**	
	The items and test exhibit good psychometric qualities and test functions as developers intended	Online testing conditions are standardised and acceptable for target population	Scoring rubric and scoring conditions are free from bias and function as intended	
**(1) Acceptance of internal-consistency reliability of test scores**	✓			
Hypothesis (1) is confirmed with final examination case scores demonstrating high internal consistency reliability with Cronbach’s alpha > 0.65 (Cronbach’s alpha 0.82 on 11-case examination)	✓			
**(2) Overall acceptable level of evidence that key-feature case scores differentiate between high- and low-achieving examinees**	✓			
Hypothesis (2.1) is accepted with item-difficulty levels between 0.2 and 0.8 in all final 11 cases	✓			
Hypothesis (2.2) is confirmed with item-total correlation of 0.30	✓			
Hypothesis (2.3) is confirmed with mean item-discrimination of 0.34	✓			
**(3) Overall acceptable level of evidence of online examination format for paediatric physicians**		✓		
Hypothesis (3.1) is confirmed. All feedback was positive regarding supported online testing format and unsupervised		✓		
Hypothesis (3.2) was rejected in 90-min examination format (70% not accepted). Plausible assumption of acceptance of final examination format with estimated time under 60-min		**?**		
(**4) Acceptance of reliability of examination scorers** and scoring rubric			✓	
Hypothesis (4) is confirmed with total congruence between masked scorers and research group > 95%			✓	
**Supporting evidence for study research questions to answer interpretation/use assumptions**	**Interpretation/ Use Argument Assumptions (as identified in **Table 2**.)**			
	**GENERALISATION INFERENCE** Accepted (✓) Partially Accepted (?) Not Accepted (x)			
	**1**	**2**	**3**	**4**
	Number of key-feature cases provide a reliable estimate of candidate performance	Key-features are representative of the examination blueprint	Key-feature case scores are influenced by prior decision-making skills training in the early diagnosis of CP	Key-feature case scores will be associated with clinical performance in diagnosing infants with CP under 6-months of age
Acceptance of reliability. Internal consistency-reliability on exam with 11 cases = 0.82	✓			
Acceptance of distribution of cases mapped to blueprint and priority behaviours		✓		
Plausibility of mean case scores increasing with years of experience in the early diagnosis of CP and prior training in early diagnosis CP diagnostic tests. Small sample size prohibits statistical analysis			**?**	
Plausible assumption but no accepted validity evidence to date				**x**
	**Interpretation/ Use Argument Assumptions (as identified in **Table 2**.)**			
**Supporting evidence for study research questions to answer interpretation/use assumptions**	**EXTRAPOLATION INFERENCES** Accepted (✓) Partially Accepted (?) Not Accepted (x)			
	**1**	**2**	**3**	**4**
	Key-feature cases test the skills essential to physician clinical decision-making in the early diagnosis of CP in a clinical setting	Key-feature cases are authentic representations of real-world cases	Key-feature case scores differentiate levels of expertise in the early diagnosis of CP	Key-feature case scores will be associated with clinical performance in diagnosing infants with CP under 6 months of age
Agreement of key-features from experts in research and clinical practice	✓			
High acceptance for authenticity, 90% of participants agreeing that cases resembled problems from clinical practice		✓		
Plausible assumption only, small sample size prohibits statistical analysis			**?**	
No accepted validity evidence to date. Further evidence required. Plausibility of methodology in future randomised controlled trial				**x**
	**Interpretation/ Use Argument Assumptions (as identified in **Table 2**.)**			
	**IMPLICATIONS INFERENCES** Accepted (✓) Partially Accepted (?) Not Accepted (x)			
**Supporting evidence for study research questions to answer interpretation/use assumptions**	**1**	**2**	**3**	
	Key-feature case scores predict or will be associated with patient outcomes of early intervention and parent supports	Development of a new key- features examination was feasible for the development group in the field of CP	Completion of the examination will have consequences for examinee candidates in creating desirable difficulties and driving learning	
Plausible assumption of physician key-feature case scores associated with external criteria of physician referrals and patient outcomes of early intervention and parent supports but no accepted validity evidence to date. Strength in national datasets for patient referrals and funding outcomes	x			
Established methodology, expert consultancy, budget to support resource development		✓		
User acceptance feedback indicative of complexity in cases and interest in feedback and learning resources			✓	

## Validity argument

An overarching validity argument was constructed through the synthesis of evidence across the chain of inferences from our specified interpretation and use of key-feature examination scores. The established validity evidence supporting assumptions and organised by each level of inference is summarised in Table 4.

### Scoring

Acceptance of scoring inferences are defensible through: (1) appraisal of empirical evidence supporting the key-features methodology measuring the construct of clinical decision-making skills; and (2) collection of prioritised new evidence through examination development, piloting and refinement phases of this study. Experts in CP and the key-features methodology followed a robust process to develop key-features cases backing the scoring assumption that the construct in the newly developed CP key-features examination measures clinical decision-making skills. Piloting of the examination with practising physicians provides sources of evidence to support psychometric test item qualities, reliability of scoring and online testing conditions. Examination pilot data provides supporting evidence for the interpretation-use argument and acceptance of pilot study hypotheses: (1) high internal consistency final examination (Cronbach’s alpha 0.82); (2) acceptable item-discrimination final examination (2.1) item-difficulty levels between 0.2 and 0.8 in all final 11 cases; (2.2) mean inter-item-correlation of 0.30; (2.3) mean item-discrimination of 0.34; (3) user acceptance (3.1) acceptance of online format through user feedback; and (4) acceptable reliability of examination assessors (total congruence masked assessor 95.2%). The scoring inference not accepted following examination piloting was (3.2) acceptability of examination time for practising physicians (70% reported not acceptable). Further sources of evidence for this assumption were prioritised in the examination refinement phase, justifying acceptance of the final examination time following a robust case item reduction process.

### Generalisation

Acceptance for generalisation inferences of influence of prior early diagnosis of CP training and years of experience in mean case scores is limited by the small sample size of pilot data, prohibiting statistical analysis. However, differences in mean case scores were observed with CP specialisation and prior training in gold standard early diagnosis of CP tests.

### Extrapolation

Partial acceptance of extrapolation inferences for the context of the use of examination scores in a future RCT to reflect clinical decision-making skills in the real-world is defensible through evidence collected during development and piloting phases: (1) rigorous research prioritisation processes to establish domains; (2) content representation to the domain of clinical decision-making skills in the early diagnosis of CP; (3) expert advisory group consensus of key-feature cases, and (4) pilot data supporting authenticity of cases (90% agreement). The accepted chain of inferences in the interpretation use argument to date provides the necessary foundation to support the plan to gather new evidence of association of examination scores with clinical performance and patient outcome measures in a future RCT.

### Implications

The progression from examination scores to assumptions about individuals’ outcomes and implications was considered from the perspectives of: (1) the physician completing the examination; (2) the infant with a CP diagnosis and their parents/carers; and (3) the key-feature examination developer. Acceptance of key-feature cases as authentic and driving interest in learning was supported with pilot physician feedback. The consequences of the examination driving learning in physicians will be considered in the RCT post-test design to reduce this validity threat. The future RCT will evaluate the association of physician examination scores with physician referrals to CP population registers and the Australian National Disability Insurance Scheme. The RCT methodology enables the collection of new validity evidence to support or refute our assumptions of association of physician examination scores with patient outcomes of age of diagnosis and early intervention and funding supports [34]. The results of this exploratory study supports the feasibility of the development of a key-features examination in the field of CP with an expert advisory group using estblished key-features methodology and expert consultancy in the key-features approach.

## Discussion

Through application of an argument-based approach, validity evidence was collected for the use of key-feature case scores as an outcome of a tailored implementation intervention for physician CP diagnosis. Feasibility of key-feature case development with CP experts was achieved. Validity evidence evaluated through examination development and piloting supports acceptance of scoring assumptions of Kane’s framework and partial acceptance of generalisation, extrapolation, and implications assumptions. Future studies will target sources of criterion relationships validity evidence to strengthen the argument for real-world performance and patient outcomes.

The high reliability achieved with low key-feature case numbers was surprising, with up to 40 cases recommended to achieve internal consistency reliability coefficient of 0.8 from previous studies [35]. Heterogeneity of pilot participants may have contributed, as identified by Trudel et al [42] in their 9-key-feature case examination with general and sub-specialty groups. However, the small sample size of our pilot limits interpretation.

Our finding of low acceptance of a 90-min 21-case examination highlights the importance of reducing the time burden for practising physicians. This result is congruent with field leader recommendations that optimisation of time is an essential consideration regarding physician participation in continuing professional development [72] and research activities [73–75]. Piloting of the examination with a small sample of the target population enabled the collection of prioritised sources of validity evidence without impacting powered RCT recruitment in the Australian context. Participation in the RCT is voluntary. Paediatrician physicians who completed the pilot key-features examination will not be eligible to participate in the RCT. Within the validity argument, trade-offs needed to be considered regarding consequences for physician participants’ study burden and psychometric perspectives to judge the level of acceptance of evidence appropriate for the purpose of continuing professional development [20]. Limitations identified with the standardised recoding of time for the online examination on the REDCap platform should also be considered in future studies evaluating consequences evidence for examinees. Suggestions from web-based eLearning evaluations may be applicable in future studies in defining thresholds for time on a page as long latency periods that may indicate absence from the platform and overestimate time spent on a question [76].

The feasibility of the assessment of physician communication skills when delivering a diagnosis using the key-features approach is significant. An increased time burden was identified for ‘write-in’ responses for communication questions by pilot participants and developers, who advised reducing sources of irrelevant variance in scoring key development. Further investigation of key-feature questions assessing communication skills is warranted.

That our pilot key-feature cases stimulated an interest in learning is not surprising as it is well accepted that assessment drives learning [77–80] and that completing an assessment can be considered an education intervention. Future exploration of the use of key-feature cases for both formative purposes in an online intervention development and summative purposes for intervention evaluation is warranted.

This study demonstrates strength in providing a worked example of a validity argument in the fields of CP, implementation science and continuing professional development outcome measures. The study methodology has potential for replication in other high-, middle- and low-income country contexts targeting adherence to clinical guidelines in CP diagnosis. This study is limited by the small pilot sample size and by not substantiating all assumptions in the interpretation use argument, however, defensible scoring evidence provides the necessary foundation for Kane’s chain of inferences and the weakest inferences identified are the primary focus in future validation studies.

## Conclusions

This study answers the call to appraise the validity evidence of health professions education and implementation instrument scores. The key-features approach shows good application in the field of CP. Argument-based validity frameworks can be applied to evaluations of health professional implementation.

### Supplementary Information

**Additional file 1: Supplementary File 1.** Key- Features Writing Fact Sheet.

**Additional file 2: Supplementary File 2.** Key-Features Writing Template.

**Additional file 3: Supplementary File 3.** Example of a cerebral palsy key-features case and questions.

**Additional file 4: Supplementary File 4.** Tables.

## Data Availability

Data that supports the findings of this study are within the article and supplementary materials. Further data is available from the corresponding author, LM, upon reasonable request.
