# Supplementary material for: Constructing validity evidence from a pilot key-features assessment of clinical decision-making in cerebral palsy diagnosis: application of Kane’s validity framework to implementation evaluations
Source: BMC Med Educ. 2023 Sep 14;23:668. doi: 10.1186/s12909-023-04631-4 (PMC10503270; doi:10.1186/s12909-023-04631-4)
Supplement: Supplementary file 1 — Additional file 1: Supplementary File 1. Key- Features Writing Fact Sheet. [file 12909_2023_4631_MOESM1_ESM.pdf]

A Key-Features Approach to Assess Clinical Decision Making

KEY FEATURES FACT SHEET
01

Developing clinical decision-making problems using the key-features approach

Key-feature cases are based on a clinical scenario with 1-3 related questions on the unique critical elements, that are essential to clinical decision making or that are often performed incorrectly in practice.[1] They have been used in physician high stakes examinations.[2] and have demonstrated construct validity sensitive to the impact of educational interventions.[3,4] Validity and reliability evidence demonstrates test elements, labelled as key-features if properly designed, can measure the construct of clinical decision making rather than knowledge. [1,3,4]

GUIDELINES FOR KEY-FEATURE CASE WRITING.[5,6]

STEP 1 – ASSEMBLE WRITING GROUPS

Include clinical multi-professional expertise from diverse metropolitan, rural and remote practices.

STEP 2 – IDENTIFY DOMAIN

- Create a focussed examination blueprint.
- Identify the target domain and learning objectives.

STEP 3 – CHOOSE CLINICAL PROBLEMS

- Choose relevant clinical problems from the domain and learning objectives and select a representative sample.
- Think about real cases from clinical practice.
- Align complexity of clinical problems with the level of learner and content area from clinical practice.
- Represent a sample of clinical problems from the target domain.

STEP 4 – DEFINE KEY-FEATURES FOR EACH CLINICAL PROBLEM

- Define a list of key-features for each problem selected from the domain.
- Determine the critical or essential clinical decision-making steps for the resolution of the clinical problem.
- Identify elements most likely to result in errors or challenging steps in diagnosing and managing the problem in clinical practice, or the common misconceptions about the clinical scenario or areas of current standard practice that are requiring quality improvement to align with evidence-based guidelines.
- Differentiate between decisions or steps that are appropriate but not critical, and the steps that are essential to identify and manage the patients problem (key-features). Discuss to achieve consensus.
- The unique key-features may pertain to history, communication, initial data gathering and diagnostic steps, other investigations, clinical decision making, immediate or longer term management, ethical and professional behaviours and prevention of complications.
- Decide if the critical steps require eliciting data, interpreting data or managing the condition. Focus on the clinical decisions or clinical actions (e.g. order an MRI) where the action is an expression of a clinical decision.
- Select the 2 to 3 most critical key features. Consider they key-feature level of difficulty appropriate for the learner.
- State the key-feature in a single sentence in lay language. The key-feature statement should contain the following parts:
  1. The initial clinical information (i.e., “Given an infant patient presenting with …, the examination candidate will”);
  2. The clinical task (e.g.., “…Order investigations including …”); and
  3. If necessary, a qualifier(s) may be added (e.g., “Order immediate ”)

For each problem and the corresponding set of key-features, include one or more references to support the key-features. References may include clinical guidelines, training materials and peer-reviewed publications.

Cerebral Palsy ALLIANCE
THE UNIVERSITY OF SYDNEY
AusCP-CTN Australasian Cerebral Palsy Clinical Trials Network CENTRE FOR RESEARCH EXCELLENCE

A Key-Features Approach to Assess Clinical Decision Making

EXAMPLE OF KEY FEATURES

1. *Integrate history and examination findings, reason diagnostically in the context of a likely benign breast lesion*
2. *Able to define / interpret imaging abnormalities in context*
3. *Apply safe clinical decision-making according to current Australian clinical guidelines for the investigation of a breast mass.*

STEP 5 – DEVELOP THE SCENARIO

- Following a discussion of the key-features select a case for development into a scenario and related questions.
- Develop an authentic clinical case scenario from real-life situations to represent the clinical problem and its key features.
- Create problems that reflect clinical practice challenges in a range of locations, age groups, and ethnic and cultural backgrounds.
- Scenario length can vary. Shorter scenarios if the key-feature is to primarily elicit data (patient demographics, limited clinical information), longer if key-features require data interpretation or management (contains laboratory and diagnostic information).
- Use of high quality visuals is encouraged. e.g. Neuroimaging.
- Use lay language and include the relevant specific case information, such as age, gender, setting, presenting condition, and any other appropriate details that enable the test taker to complete the answers in a focused and specific manner.

EXAMPLE OF A SCENARIO

You are a family doctor. Ms Jane McNamara aged 25, has come to see you last week about a small lump in her right breast that she noticed in the shower. At that time she was post menstrual. The lump was not tender and she had never noticed anything like that before. She's taking the oral contraceptive pill, her periods are regular and she has never been pregnant.

On examination there was a discrete small mass in the right lower quadrant, which was not tender and somewhat mobile. It was approximately 1.5 cm in diameter.

There was no other abnormality on examination of the breast or the contralateral breast. Both axillae where normal.

The rest of her history and examination was unremarkable. There was no family history of breast malignancy known to the patient.

You referred Jane for an ultrasound of the breast and she has returned for review with the ultrasound images.

STEP 6 – DEVELOP THE QUESTIONS

- Write questions that test the key-features. Most case scenarios are followed by 2 or 3 questions.
- Select the appropriate question format from write-in (examinees supply their responses in note form only, not sentences, e.g. order MRI) short-menu (select from a prepared list, e.g. high-risk of cerebral palsy).
- Focus the question and use specific qualifiers. e.g. What element(s) of the history if any, are most important to gather (ask about; elicit, focus on) or are most likely to confirm your diagnosis?
- The qualifier may reflect the urgency or a decision e.g. What initial action…?, or a decision-making priority, e.g. What are the most important…?
- The question stem consists of a direct formulation in clear phrasing. e.g. What are the most important/ relevant items of information you will give to the patient? What key investigations would you order at this consultation?
- The questions derive directly from the key-features and focus exclusively on clinical actions or decisions as opposed to the reasoning behind the action. e.g. What investigation(s) [test(s)], if any, will you order at this point?
- Generally, one question tests one key-feature but can occasionally test more than one key-feature if appropriate. e.g. What management option(s) will you offer [recommend to] your patient at that time?
- Avoid questions that can be answered without reference to the clinical scenario as these may not be measuring clinical decision-making. The questions must follow from the scenario.
- It is useful to include a summary table illustrating the relationship between the test question and key-features.

A Key-Features Approach to Assess Clinical Decision Making

	KF1	KF2	KF3
Q1	x		
Q1		x	x
Q1			

Question one (diagnostic reasoning question)

What are the most likely initial diagnoses? List in note form only up to two diagnoses.
1
2

Question two (interpretation of tests in context, familiarity with the imaging mode)

What key features are shown on this image? List in note form only up four key features.
1
2
3
4

Question three (clinical decision regarding immediate management must be according to guidelines (e.g. triple test)

What are the most important immediate next steps? List in note form only up to 3 next steps.

1
2
3

STEP 7 – DEVELOP THE ANSWERS

- Develop clear instructions for answering each question, including the number of permissible responses, e.g. " list up to four."
- If the question is not the first in a series of questions pertaining to the clinical scenario, it may also contain additional information, e.g. " The patient has now had the following investigations______ what further assessments would you now recommend?"
- Choose your response format of short-answer "write-in" responses (examinees supply their responses, e.g. order MRI) or "short-menu" responses (select from a prepared list, e.g. high-risk of cerebral palsy).
- The number on the short menu list can range from 5 to as many as 30 if required. Keep the list length plausible to real-life options.
- Lists should be presented in alphabetical order, the same length and grammatical structure.
- The list should contain all possible correct responses (there can be more than one) and any incorrect answers.
- Provide two incorrect options for each correct option on the list.
- At the end of each list, the last option will be "none" meaning that no action is needed at that particular time.
- Write-in responses should be limited to short notes or single words.

STEP 8 – DEVELOP THE SCORING GUIDELINE

- The scoring key consists of the list of correct responses to a key-feature question and a system of assigning numerical scores to the answers.
- The correct scored responses should directly reflect the key-features being assessed.
- Each key-feature question within a case is usually given a maximum score of "1" and a minimum score of "0". " If one action is considered much more important than another, the answer score may be weighted to reflect its importance. Weighting should be simple e.g. normally a score of 2 or at the most a score of 3.
- Key-feature questions usually have more than one correct answer, which is a significant benefit of this assessment style. A summed and partial credit scoring system can be used.[3]
- Negative marking (if a harmful or unnecessary investigation was ordered, or prescribing inappropriate interventions) has more recently fallen out of favour but it may be considered.[3]
- Equal weights of each key-feature within each case can be averaged to generate a key-feature case score.
- A total examination score will be calculated by averaging key feature case scores across the test.
- They represent the average proportion /percentage of key features mastered for each case on the examination.[3]

1. Page G., Bordage., Allen T. Developing key-feature problems and examinations to assess clinical decision-making skills.(1995) Academic Medicine 1995; 70(3):194-201.
2. Page G, Farmer E, Spike N, McDonald E. The use of short answer questions in the key features problems in the Royal College of General Practitioners Fellowship examination. Abstract. In: Proceedings of the 10th Ottawa Conference Cape Town, South Africa, 2000. Combining marks, scores and grades.
3. Bordage G., Page G. The key-features approach to assess clinical decisions: validity evidence to date. Adv in Health Sci Educ 2018:23:1005-1036.
4. Hrynchak, P, Takahashi S, Nayer M. Key-feature questions for assessment of clinical reasoning: a literature review. Medical Education 2014: 48: 870–883 doi: 10.1111/medu.12509
5. Medical Council of Canada. GUIDELINES FOR THE DEVELOPMENT OF KEY FEATURE PROBLEMS & TEST CASES August 2012 (v3) https://mcc.ca/ media/CDM-Guidelines.pdf
6. Farmer, E. A. and Page, G. (2005). A practical guide to assessing clinical decision-making skills using the key features approach. Medical Education, 39: 1188-1194. doi:10.1111/j.1365-2929.2005.02339.x
7. Page GG, Bordage G. The Medical Council of Canada's Key Features Project: a more valid written examination of clinical decision-making skills. Acad Med 1995; 70: 104 – 10
8. America Education Research Association. American Psychological Association and National Council on Measurement and Education. Standards for educational and psychological testing Washington DC: American Educational Research Association. 2014.
9. Lane S, Raymond MR, Haladyna TM. Handbook of Test Development.2nd ed. New York., NY; Routledge; 2004.
10. Haladyna TM, Rodriguez Mc. Developing and Validating Test Items. New York, NW. Routledge; 2013.
11. Bordage G, Brailovsky C, Carretier et al. Content validations of key features on a national examination of clinical decision-making skills. Acad Med 1995; 70:276-281.
12. Sullivan M, Park YS, LiscumK, Sachdeva K, Blair PG, Gesbeck M, Bordage G. The American College of Surgeons Entering Resident Readiness Assessment Program. Annals of Surgery. 2019
13. Lang V, Berman N, Bronander K, et al. Validity Evidence for a Brief Online Key Features Examination in the Internal Medicine

Examples of a Key-Feature case and questions

Example One. Written by Professor Elizabeth Farmer for educational purposes.

Key features

1 Integrate history and examination findings, reason diagnostically in the context of a likely benign breast lesion
2. Able to define / interpret imaging abnormalities in context
3. Apply safe clinical decision-making according to current Australian clinical guidelines for the investigation of a breast mass.

Case

You are a family doctor. Ms Jane McNamara aged 25, has come to see you last week about a small lump in her right breast that she noticed in the shower. At that time she was post menstrual. The lump was not tender and she had never noticed anything like that before. She's taking the oral contraceptive pill, her periods are regular and she has never been pregnant.

On examination there was a discrete small mass in the right lower quadrant, which was not tender and somewhat mobile. It was approximately 1.5 cm in diameter.

There was no other abnormality on examination of the breast or the contralateral breast. Both axillae were normal.

The rest of her history and examination was unremarkable. There was no family history of breast malignancy known to the patient.

You referred to Jane for an ultrasound of the breast and she has returned for review with the ultrasound images.

Question one (diagnostic reasoning question)

What are the most likely initial diagnoses? List in note form only up to two diagnoses.
1
2

You review the ultrasound. (follow on - no further clinical information required)

Question two (interpretation of tests in context, familiarity with the imaging mode)

What key features are shown on this image? List in note form only up four key features.
1
2
3
4

Question three (clinical decision regarding immediate management must be according to guidelines (eg triple test)

What are the most important Immediate next steps? List in note form only up to 3 next steps.

1
2
3

A Key-Features Approach to Assess Clinical Decision Making

KEY FEATURES FACT SHEET 03

Examples of key-feature test questions

History & physical examination

- What [pertinent] clinical information are most important to elicit at this time?
- As you review the chart and take their history, what information is most likely to assist your management?
- What else, if any, will you ask him [her] at this time in order to xxx?

Diagnosis

- What is your leading [working] diagnosis at this point? List only one.
- What are the most likely/important/relevant diagnosis(es) you are considering [thinking about] at this time? List up to three.
- The result is now available (show film of …). Identify up to xx abnormalities or features. What is your diagnosis?

Investigation

- What investigation(s) [test(s)], if any, are most relevant/important to order at this point?
- What are the most relevant/important test(s), if any, that you will order next?
- What further [additional] investigation(s), if any, are the most important or most relevant for you order?

Management

- What essential steps will you take in your immediate assessment and management of this patient?
- What treatment [measures] [additional measures] will you recommend today? Be specific.
- What will you recommend as the most important or most relevant next step(s) in the management of this patient?

Response limits

Every question is followed by one of the following statements that sets the limit of allowable responses:

☐ **List (select) only one.**
This type of limit is suitable for requesting a single definitive answer, such as a leading diagnosis or the most important management step.

☐ **List (select) up to x.**
This type of limit is suitable for questions in which one or more answers are sought and for which the number of opportunities for examinees to provide these answers should be capped (i.e., forcing the issue). The "x" (number of allowable responses) takes into account the number of correct keyed responses sought, together with other factors such as responses that would be reasonable but not in the keyed responses as well as incorrect responses that might be appealing to weaker candidates.

☐ **List (select) as many as are appropriate.**
This type of limit is used in situations where it is useful to determine how many actions an examinee might take, as in the case of limiting the investigation or treatment (i.e., not over investigating or treating). The maximum number of responses is specified in the scoring key, but the examinees are blinded to this limit.

Candidates are warned about these different types of limits in the general instructions to the exam.

Medical Council of Canada. GUIDELINES FOR THE DEVELOPMENT OF KEY FEATURE PROBLEMS & TEST CASES August 2012 (v3) https://mcc.ca/media/CDM-Guidelines.pdf
