# Supplementary material for: Constructing validity evidence from a pilot key-features assessment of clinical decision-making in cerebral palsy diagnosis: application of Kane’s validity framework to implementation evaluations
Source: BMC Med Educ. 2023 Sep 14;23:668. doi: 10.1186/s12909-023-04631-4 (PMC10503270; doi:10.1186/s12909-023-04631-4)
Supplement: Supplementary file 2 — Additional file 2: Supplementary File 2. Key-Features Writing Template. [file 12909_2023_4631_MOESM2_ESM.pdf]

KFP Template

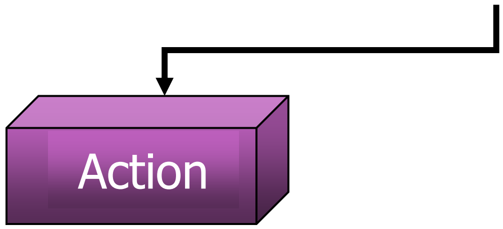

Author(s)

Case name

Key features

Main Scenario

Question 1

Answer 1: Write in (1-5) Selection (1-20)

Identify correct answers

1	11
2	12
3	13
4	14
5	15
6	16
7	17
8	18
9	19
10	20

Extra scenario?

Question 2

Answer 2: Write in (1-5) Selection (1-20)

Identify correct answers

1	11
2	12
3	13
4	14
5	15
6	16
7	17
8	18
9	19
10	20

Extra scenario?

Question 3

Answer 3: Write in (1-5) Selection (1-20)

Identify correct answers

1	11
2	12
3	13
4	14
5	15
6	16
7	17
8	18
9	19
10	20

Notes
