# Supplementary material for: Constructing validity evidence from a pilot key-features assessment of clinical decision-making in cerebral palsy diagnosis: application of Kane’s validity framework to implementation evaluations
Source: BMC Med Educ. 2023 Sep 14;23:668. doi: 10.1186/s12909-023-04631-4 (PMC10503270; doi:10.1186/s12909-023-04631-4)
Supplement: Supplementary file 3 — Additional file 3: Supplementary File 3. Example of a cerebral palsy key-features case and questions. [file 12909_2023_4631_MOESM3_ESM.pdf]

Supplementary File 3.

KEY FEATURES

Given a term newborn with lateralized clonic seizures at birth, with no other signs of neonatal encephalopathy, and ultrasound at 3 days showing no clear damage, the candidate will consider performing magnetic resonance imaging (MRI) to explore the diagnosis of unilateral damage (Key feature 1). As the MRI is performed at 3 days of life, the candidate will consider asking for a diffusion sequence on MRI (Key feature 2). The MRI shows clear damage to the left hemisphere, especially on the diffusion weighted images (DWI). However, there are no clear signs of asymmetry in the posterior limb of internal capsule (PLIC). The candidate will then suggest performing the General Movements Assessment (GMA) at fidgety age (Key feature 3).

MAIN SCENARIO

Anna was born at 39 weeks' gestation.

At the moment of discharge, the nurse noted some repetitive limb movements on the right side. Anna had an EEG on day 2, but during the recording the movements were not seen. The EEG failed to show clear asymmetries.

She also had a head ultrasound that did not show clear signs of brain damage.

Anna is now 3 days old.

QUESTION 1

What is the most important diagnostic assessment to order at this point?

Identify the correct answer from the list.

Select only one (1) answer from the list.

ANSWER 1

Scoring Key

Answer	Score
**Maximum score for Question 1**	**1**
1. Assessment of Visual Functions	**0**
2. Brain MRI	**1**
3. Cranial US	**0**
4. EEG	**0**
5. General Movements Assessment (GMA)	**0**
6. Hammersmith Infant Neurological Examination (HINE)	**0**
7. Hip X-ray	**0**
8. None	**0**

QUESTION 2

An MRI is planned for day 3 of life.

What type of MRI acquisition would you make sure is performed?

Select one (1) single answer from the list.

ANSWER 2

Answer	Score
**Maximum score for Question 2**	**1**
1. Diffusion weighted imaging DWI	**1**
2. EEG/MRI	**0**
3. MR Spectroscopy	**0**
4. Resting state	**0**
5. Standard T1 and T2 are enough	**0**

QUESTION 3

The MRI shows clear damage to the left hemisphere, especially on the DWI images. There are no clear signs of asymmetry in the Posterior Limb Internal Capsule (PLIC).

What is the most important next step in your management?

Select only one (1) single answer from the list.

ANSWER 3

**Answer**	**Score**
1. Schedule an appointment as soon as possible to perform General Movements Assessment (GMA)	**1**
2. Schedule an appointment at 6 months to perform Hammersmith Infant Neurological Examination (HINE)	**0**
3. Refer the patient to early intervention	**0**
4. Communicate to the parents the high risk of unilateral cerebral palsy	**0**
5. Wait and see	**0**
**Maximum score for Question 3**	**1**
