# Supplementary material for: Constructing validity evidence from a pilot key-features assessment of clinical decision-making in cerebral palsy diagnosis: application of Kane’s validity framework to implementation evaluations
Source: BMC Med Educ. 2023 Sep 14;23:668. doi: 10.1186/s12909-023-04631-4 (PMC10503270; doi:10.1186/s12909-023-04631-4)
Supplement: Supplementary file 4 — Additional file 4: Supplementary File 4. Tables. [file 12909_2023_4631_MOESM4_ESM.pdf]

Supplementary File 4

Table 1. Demographic characteristics of pilot study participants (n=10)

**Characteristics**	**Categories**	**Percentage (%)**	**Notes**
Practice type	Rehabilitation specialist	40	
	Paediatrician	80	3 participants paediatrician + rehab specialist
	Neonatologist	10	1 participant both paediatrician + neonatologist
	Paediatric registrar	10	
Number of years working	> 20 years	40	
	> 10 years	30	
	5 - 10 years	30	
Number of years' experience diagnosis of cerebral palsy	> 20 years	20	
	10 - 20 years	40	
	5 - 9 years	10	
	< 5 years	20	
	Never	10	
Current workplace	Tertiary hospital	40	1 participant both tertiary + community health
	Hospital other - metro, regional, rural	50	1 participant both hospital other + private
	Community health	10	
	Private practice	20	1 participant both hospital other + private
Proportion of caseload cerebral palsy	51 - 75%	30	
	0 -10%	70	
Awareness of Novak et al 2017 clinical guideline[30]	Yes	60	
	No	40	
HINE and GMA training	Yes	30	
	No	70	

Abbreviations: HINE=Hammersmith Infant Neurological Examination; GMA=Prechtl's General Movements Assessment; Guidelines=Novak et al (2017); CP=cerebral palsy; rehab=rehabilitation

Table 2. Relative distribution of cases in 21-case examination according to blueprint

	No. Cases	% Cases	No. Key Features	% Key Features
Cerebral palsy type topography severity	6	28.6	6	9.5
Cerebral palsy risk factors	10	47.6	10	15.9
General Movements Assessment	16	76.2	21	33.3
Hammersmith Infant Neurological Examination	15	71.4	25	39.7
Communication skills for providing diagnosis	11	52.4	15	23.8
Neuroimaging	18	85.7	27	42.9
Early intervention	7	33.3	9	14.3

Table 3. Pilot study mean case scores according to awareness of clinical guideline, clinical caseload, and prior training (n=10)

	Mean	Mean
Mean case scores according to awareness of guidelines	Yes	No
	12.98	9.95
Mean case scores according to proportion of caseload CP	50 - 75%	0 - 10%
	12.59	9.99
Mean case scores prior HINE + GMA training	GMA + HINE	No GMA or HINE
	13.48	11.47

Abbreviations: SD=Standard Deviation; HINE=Hammersmith Infant Neurological Examination; GMA=Prechtl's General Movements Assessment; Guidelines=Novak et al (2017); CP=cerebral palsy

Table 4. Relative distribution of cases in 11-case examination according to blueprint

	No. Cases	% Cases	No. Key Features	% Key Features
Cerebral palsy type topography severity	3	27.3	3	11.1
Cerebral palsy risk factors	4	36.4	4	14.8
General Movements Assessment	9	81.8	9	33.3
Hammersmith Infant Neurological Examination	9	81.8	14	51.9
Communication skills for providing diagnosis	4	36.4	5	18.5
Neuroimaging	9	81.8	14	51.9
Early intervention	4	36.4	5	18.5
